# Correlation between PCT, 25(OH)D, PTX-3, AMS levels and the severity of diabetic ketoacidosis complicated by pancreatitis

**DOI:** 10.1186/s12902-021-00792-9

**Published:** 2021-06-29

**Authors:** Songtao Lu, Dongmei Wei, Chao Yin, Juwen Xiong, Lishuang Zhu, Shaoru Yan, Rui Meng

**Affiliations:** 1 Department of Geriatrics, Tangshan Worker Hospital, No. 27 Wenhua Road, Lubei District, Tangshan, 063000 China; 2 Department of Emergency, Tangshan 120 Emergency Command Center, Tangshan, China; 3 Department of Rehabilitation, Tangshan Worker Hospital, Tangshan, China; 4 Department of Orthopaedics, Tangshan People’s Hospital, Tangshan, China; 5 Department of Rheumatology and Immunology, Tangshan Worker Hospital, Tangshan, China

**Keywords:** Procalcitonin, 25-hydroxyvitamin D3, Pentraxin-3, Amylase, Diabetic ketoacidosis, Pancreatitis, Severity of illness

## Abstract

**Background:**

This study aims to explore the correlation between procalcitonin (PCT), 25-hydroxyvitamin D3 (25(OH)D), pentraxin-3 (PTX-3), amylase (AMS) levels and severity of diabetic ketoacidosis complicated by pancreatitis.

**Methods:**

A retrospective analysis of 198 patients with diabetic ketoacidosis admitted to our hospital from January 2015 to February 2020 were included. According to whether the patients with pancreatitis, subjects were divided into diabetic ketoacidosis with pancreatitis (DKA-AP) group and diabetic ketoacidosis (DKA) group. Healthy controls admitted to the hospital for physical examinations were included as a control group. Clinical outcomes were collected.

**Results:**

On the first day after admission, the levels of PCT, PTX-3, and AMS in DKA-AP group were significantly higher than those in DKA group and control group, and 25(OH)D levels in DKA-AP group were lower than those in DKA group and control group. PCT, PTX-3, and AMS levels were significantly increased, and 25(OH)D levels were decreased in the DKA group compared with the control group. Furthermore, the levels of PCT, 25(OH)D, PTX-3, and AMS in the DKA-AP group were correlated with the disease severity of of diabetic ketoacidosis complicated by pancreatitis. The levels of PCT, PTX-3, and AMS in the DKA-AP group on day 1 were significantly higher and 25(OH)D levels were significantly lower than those on days 3–7 after admission. The levels of PCT, PTX-3, and AMS in the DKA group on day 1 were significantly higher and 25(OH)D levels were significantly lower than those on days 2–7 after admission. The levels of these indicators returned to normal levels on day 3 or day 7 in DKA or DKA-AP group, respectively. PCT, PTX-3, and AMS levels in the DKA-AP group were significantly increased, while 25(OH)D levels in the DKA-AP group were decreased compared with DKA group on days 1–6 after admission. The duration of hospital stay, patients of ICU care, duration of ICU stay, and cost in DKA-AP group were all higher than those in the DKA group.

**Conclusion:**

Blood levels of PCT, 25(OH)D, PTX-3, and AMS were correlated with diabetic ketoacidosis complicated by pancreatitis, and have certain application value in assessment of the disease severity.

## Background

Diabetic ketoacidosis is one of the common acute complications of diabetes mellitus, accompanied by increased ketones in the blood, metabolic disorders, and metabolic acidosis [1, 2]. Studies have shown that about 11% of diabetic ketoacidosis is complicated by pancreatitis, leading to increased incidence of multi-organ failure and mortality and aggravating the disease severity [3]. Early diagnosis and treatment are of great value and significance to these patients. In clinical practice, the diabetic ketoacidosis with pancreatitis is diagnosed by a comprehensive analysis of clinical indicators (including acute physiology and chronic health evaluation II (APACHE II) and Ranson scores), laboratory tests and imaging examinations. However, APACHE II and Ranson scores are complicated and tedious, which require a lot of time to acquire. It has been reported that inflammatory factors such as tumor necrosis factor, interleukin (IL) -6, IL-8 and other cytokines are associated with diabetic ketoacidosis accompanied by pancreatitis, while their sensitivity and specificity are not ideal [4]. Therefore, effective indexes are urgently needed to accurately assess the condition of diabetic ketoacidosis complicated by pancreatitis, which could guide clinicians to conduct effective medical treatments.

It is reported that procalcitonin (PCT), 25-hydroxyvitamin D3 (25(OH)D), pentraxin-3 (PTX-3), and amylase (AMS) are potential biomarkers for diabetic ketoacidosis or pancreatitis. PCT is associated with multiple organ failure, sepsis, and serious infections [5], and which is significantly increased in patients with diabetic ketoacidosis [6]. Hiba et al. found that 25(OH)D was lower in type I diabetes (T1D) patients, especially those with diabetic ketoacidosis [7]. Moreover, patients with moderate-to-severe acute pancreatitis also have decreased 25(OH)D levels [8]. PTX-3 can serve as an independent predictor of systemic infections such as sepsis [9], and PTX-3 levels are rapidly increased in the early stage of pancreatitis [10]. Patients with pancreatitis or diabetic ketoacid usually have higher concentrations of AMS [11]. However, there is no study regarding the applicability of PCT, 25(OH)D, PTX-3, and AMS in diabetic ketoacidosis patients with pancreatitis.

Herein, this study aimed to investigate the correlation between the levels of PCT, 25(OH)D, PTX-3, AMS, and the severity of diabetic ketoacidosis with pancreatitis, which may provide a theoretical basis for clinical treatment.

## Methods

### Participants

The retrospective study was conducted in our hospital. All patients (198 cases with 86 males and 112 females) with diabetic ketoacidosis admitted to the hospital from January 2015 to February 2020 were identified. There were 37 subjects with pancreatitis and 161 subjects were not.

### Ethics

Ethics approval and consent to participate: This research was approved by the Ethics Committee of Tangshan Worker Hospital and was conducted following the ethical standards of the Helsinki Declaration. Written informed consent was obtained from all the study subjects before enrollment.

### Inclusion, exclusion, and diagnostic criteria

Inclusion criteria: ① T1D and type II diabetes (T2D) adult patients with confirmed diabetes mellitus; ② complete clinical data; ③ obtained the informed consent of patients. Exclusion criteria: ① accompanied by serious diseases of other systems (such as heart, liver, and kidney), other acute abdominal diseases, infectious diseases of other systems, respiratory diseases, schizophrenia, chronic pancreatitis, macroglobulinemia, or concomitant malignant tumors; ② a history of drug dependence or alcoholism; ③ recent history of severe trauma or surgery.

Healthy controls were people admitted to the hospital during the study period for medical examination. According to whether the patients with or without diabetic ketoacidosis or pancreatitis, they were divided into diabetic ketoacidosis with pancreatitis (DKA-AP) group, diabetic ketoacidosis without pancreatitis (DKA) group and healthy control (Control) group.

Diagnostic criteria:

①Diabetes mellitus: according to the diagnostic criteria for diabetes mellitus established by the World Health Organization [12]. ②Diabetic ketoacidosis was diagnosed by the laboratory tests [13]: venous glucose > 11.1 mmol/L, HCO_3_^−^ < 15 mmol/L or artery blood pH < 7.3, ketonuria and ketonemia. Grade of diabetic ketoacidosis: severe: HCO_3_^−^ < 5 mmol/L or artery blood pH < 7.1; moderate: HCO_3_^−^ < 10 mmol/L or artery blood pH < 7.2; mild: HCO_3_^−^ < 15 mmol/L or artery blood pH < 7.3. ③Pancreatitis [5]: the levels of ≥2 types pancreatic enzymes were 3 times higher than normal levels and accompanied by pancreatic CT diagnosis; if the level of lipase or amylase was 3 times higher than normal levels, but there is no change in pancreatic CT, it is diagnosed with suspected pancreatitis.

### Data collection

The available information was collected from patients, their families, physician, nurses, and electronic medical records. ① Basic characteristics: sex, age, body mass index (BMI). ② Biochemical parameters: creatine, hemoglobin A_1C_ (HbA_lC_), blood urea nitrogen (BUN), low-density lipoprotein (LDL), high-density lipoprotein (HDL), aspartate aminotransferase (AST), alanine aminotransferase (ALT), carbon dioxide combining power (CO_2_CP), blood glucose, blood potassium, blood sodium, blood ketone, high sensitivity cardiac troponin testing (hs-cTnT), brain natriuretic peptide (BNP), triacylglyceride (TG), total cholesterol (TC), lactate, pH, PCT, 25(OH)D, PTX-3, AMS. All biochemical parameters were detected in venous blood. ③ Frequency of diabetic ketoacidosis, onset age of diabetes, type of diabetes, duration of diabetes, and abdominal CT. ④ Complications: microangiopathy (such as diabetic retinopathy and diabetic nephropathy) and macroangiopathy (such as peripheral arterial embolism and cardiovascular diseases). ⑤ Clinical outcomes: duration of hospital stay, patients with intensive care unit (ICU) care, duration of ICU stay, mortality, and cost.

The blood samples for detecting the biochemical parameters were collected every day in the first 7 days after admission. And the detection methods of PTC, PTX-3, 25 (OH) D, and AMS were as follows:

- PTC: PCT was detected by electrochemiluminescence.
- PTX-3: PTX-3 was detected by enzyme linked immunosorbent assay (ELISA), and the kits were purchased from Shanghai Hengyuan Biotechnology Co., Ltd.

## 25 (OH) D: 25 (OH) D was detected by ELISA, and the kits were purchased from Roche (Switzerland)

AMS: The level of AMS was detected by glucose oxidase method.

### Treatment

The patients in the DKA-AP group and DKA group received fasting, water deprivation, continuous gastrointestinal decompression, acid suppression, anti-inflammatory, and other conventional treatment after admission. After diagnosis, the patients were given continuous intravenous insulin infusion (0.1 U / (kg h), once every 4–6 h). Blood glucose and ketosis were detected regularly. Once the ketone body turned negative, the patients were treated with insulin (0.02 U / (kg · h)).

### Statistics

All the data collected in this study were analyzed using SPSS 22.0 software. Normally distributed measurement data were expressed as mean ± standard deviation (SD), while non-normally distributed measurement data were expressed as median (interquartile range), and the comparisons were examined by student *t*-test and one-way ANOVA test (normally distribution), and Mann-Whitney test (non-parametric distribution). The categorical data were expressed as n (%), and the differences between the two groups were examined by chi-square analysis or Fisher’s exact test. Pearson correlation was used to describe the relationship between 2 variables. *P* < 0.05 was considered statistically significant.

## Results

### Study population

From January 2015 to February 2020, there were 72 cases of healthy control and 198 cases of diabetic ketoacidosis patients (37 cases in DKA-AP group, 161 cases in DKA group) admitted to our hospital. There were 16 males and 21 females with the average age of 63.29 ± 6.72 years old and the average BMI of 24.38 ± 3.01 kg/m^2^ in the DKA-AP group. There were 70 males and 91 females with the average age of 62.98 ± 7.01 years old and the average BMI of 24.19 ± 2.78 kg/m^2^ in the DKA group. There were 17 cases of T1D, 19 cases of T2D, and one case of undefined type of Diabetes in the DKA-AP group, while there were 85 cases of T1D, 69 cases of T2D, and 17 cases of undefined type of Diabetes in the DKA-AP group.

There was no significant difference among DKA-AP, DKA, and control groups in sex, age, BMI, LDL, blood sodium, onset age of diabetes, and delirium (*P* > 0.05) (Table 1). The levels of creatine, HbA_lc_, BUN, HDL, AST, ALT, CO_2_CP, blood glucose, blood potassium, blood ketone, hs-TnT, BNP, TG, TC, lactate, pH, and frequency of diabetic ketoacidosis, type of diabetes, duration of diabetes, vascular complications and abdominal pain were significantly different among the 3 groups (*P* < 0.05) (Table 1). Furthermore, the differences between DKA-AP and DKA groups were not significant in sex, age, BMI, HbA_lc_, CO_2_CP, blood sodium, blood ketone and TG (*P* > 0.05). There were significant differences in blood levels of BUN, HDL, AST, ALT, blood glucose, blood potassium, hs-TnT, BNP, TC, lactate and pH, and frequency of diabetic ketoacidosis between the two groups (*P* < 0.05) (Table 1).

**Table 1:** Population characteristics

Characteristics	DKA-AP (*n* = 37)	DKA (*n* = 161)	Control (*n* = 72)	χ^2^/Z	*P*
Sex, n (%)					
Female	21 (56.76)	91 (56.52)	38 (52.78)	0.307	0.856
Male	16 (43.24)	70 (43.48)	34 (47.22)		
Age, year	63.29 ± 6.72	62.98 ± 7.01	64.01 ± 6.92	0.346	0.708
BMI, kg/m^2^	24.38 ± 3.01	24.19 ± 2.78	24.09 ± 2.68	0.267	0.766
Biochemical parameters					
Creatine, μmol/L	118.76 ± 18.73	117.98 ± 13.29	56.98 ± 6.93	391.612	< 0.001
HbA_lc_, %	9.01 ± 2.02	8.91 ± 1.97	5.37 ± 0.76	110.988	< 0.001
BUN, mmol/L	5.74 ± 0.34	5.43 ± 0.31	5.01 ± 0.28	130.692	< 0.001
LDL, mmol/L	3.02 ± 0.76	2.99 ± 0.26	2.87 ± 0.27	1.431	0.241
HDL, mmol/L	1.05 ± 0.21	0.92 ± 0.18	0.68 ± 0.23	76.904	< 0.001
AST, U/L	56.83 ± 8.92	29.98 ± 2.19	28.08 ± 2.01	458.502	< 0.001
ALT, U/L	63.19 ± 7.62	27.99 ± 2.23	27.23 ± 1.87	996.273	< 0.001
CO_2_CP, mmol/L	30.08 ± 2.72	29.76 ± 1.08	28.08 ± 1.03	9.296	< 0.001
Blood glucose, mol/L	13.02 ± 2.18	10.76 ± 1.92	5.36 ± 1.29	390.662	< 0.001
Blood potassium, mmol/L	4.38 ± 0.28	4.26 ± 0.23	4.01 ± 0.19	53.713	< 0.001
Blood sodium, mmol/L	147.93 ± 32.09	144.99 ± 31.29	142.97 ± 29.84	0.659	0.518
Bloog ketone, M (P_25_, P_75_), mmol/L	4.83 (3.47, 6.03)	0.06 (0, 0.20)	0.03 (0, 0.18)	69.876	< 0.001
hs-TnT, M (P_25_, P_75_), μg/L	0.08 (0.03, 0.019)	0.02 (0.02, 0.03)	0.01 (0, 0.14)	31.876	< 0.001
BNP, ng/L	85.43 ± 6.38	67.98 ± 8.27	54.39 ± 5.83	616.474	< 0.001
TG, mmol/L	2.73 ± 0.63	2.69 ± 0.59	2.51 ± 0.48	3.562	0.03
TC, mmol/L	5.17 ± 0.47	4.82 ± 0.39	4.51 ± 0.23	66.639	< 0.001
Lactate, M (P_25_, P_75_), mmol/L	2.71 (1.06, 4.73)	0.91 (0.51, 1.69)	0.89 (0.50, 1.61)	56.543	< 0.001
pH	7.4 ± 0.26	7.1 ± 0.23	7.0 ± 0.09	84.499	< 0.001
Frequency of diabetic ketoacidosis, M (P_25_, P_75_)	2 (1, 3)	0 (0, 1)	0	10.987	< 0.001
Onset age of diabetes, M (P_25_, P_75_), year	35 (26, 58)	36 (28, 59)	0	−0.519	0.606
Type of diabetes, n (%)					
Type I	17	85	0	12.923	< 0.001
Type II	19	41	0		
Unkown	1	35	0		
Duration of diabetes, M (P_25_, P_75_), year	9 (0.6, 19)	0 (0, 1)	0	8.876	< 0.001
Complications, n (%)					
Vascular complications	17 (45.95)	31 (19.25)	0	11.671	0.001
Abdominal pain	33 (81.08)	22 (13.66)	0	85.542	< 0.001
Delirium	2 (5.41)	14 (8.70)	0	0.438	0.508

*ALT* Alanine aminotransferase, *AST* Aspartate aminotransferase, *BMI* Body mass index, *BNP* Brain natriuretic peptide, *BUN* Blood urea nitrogen, *CO*_*2*_*CP* Carbondioxide combining power, *HbA*_*lc*_ Hemoglobin A_1C_, *HDL* High density lipoprotein, *hs-cTnT* High sensitivity cardiac troponin testing, *LDL* Low density lipoprotein, *TC* Total cholesterol, *TG* Triacylglyceride

### The levels of PCT, 25(OH)D, PTX-3 and AMS among groups

On the first day after admission, the levels of PCT, PTX-3, and AMS in DKA-AP group were significantly higher compared with DKA or control group, and the 25(OH)D level of DKA-AP group was lower than that in DKA group (*P* < 0.05) (Table 2). The levels of PCT, PTX-3, and AMS of the DKA group were significantly higher than those in the control group, while 25(OH)D level was lower compared with the control group (*P* < 0.05) (Table 2).

**Table 2:** The levels of PCT, 25(OH)D, PTX-3 and AMS among groups

Group	PCT, mg/L	25(OH)D, ng/mL	PTX-3, ng/mL	AMS, mmol/L
DKA-AP (*n* = 37)	1.32 ± 0.32^#*^	3.17 ± 0.34^#*^	10.52 ± 0.64^#*^	398.76 ± 26.65^#*^
DKA (*n* = 161)	0.87 ± 0.15^*^	15.87 ± 0.86^*^	0.34 ± 0.06^*^	107.65 ± 12.87^*^
Control (*n* = 72)	0.21 ± 0.08	30.65 ± 1.32	0.29 ± 0.04	45.87 ± 8.76
Z	609.246	366.05	707.278	571.808
*P*	< 0.001	< 0.001	< 0.001	< 0.001

*AMS* Amylase, *PCT* Procalcitonin, *PTX-3* Pentraxin-3, *25(OH)D* 25-hydroxyvitamin D3. ^#^, compared with the level on day 1 after admission, *P* < 0.05; ^*^, compared with DKA group, *P* < 0.05

### Association between potential biomarkers (PCT, 25(OH)D, PTX-3, AMS) and severity of pancreatitis complicated by diabetic ketoacidosis

**Fig. 1:**
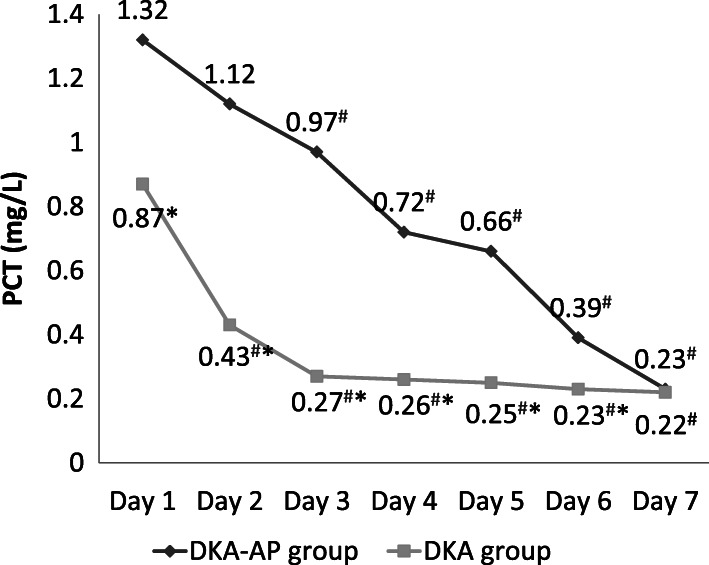
Levels of PCT in DKA-AP and DKA groups. ^#^, compared with the level on day 1 after admission, *P* < 0.05; ^*^, compared with DKA group, *P* < 0.05. PCT, procalcitonin

**Fig. 2:**
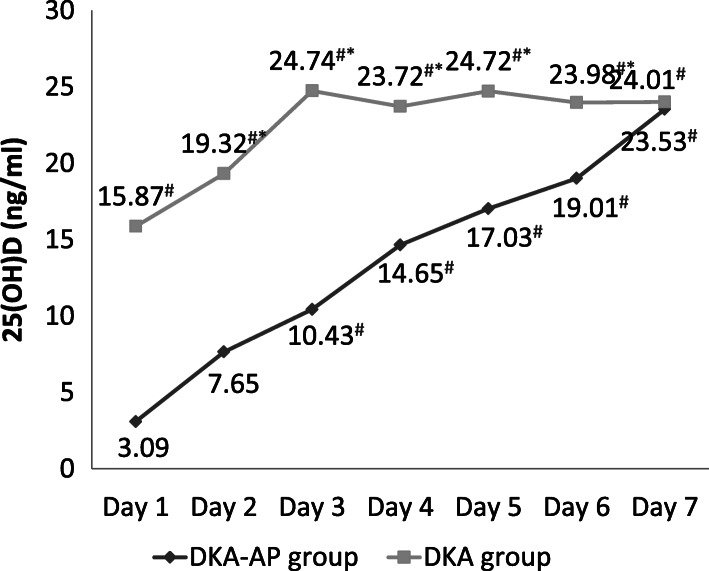
Levels of 25(OH)D in DKA-AP and DKA groups. ^#^, compared with the level on day 1 after admission, *P* < 0.05; ^*^, compared with DKA group, *P* < 0.05. 25(OH)D, 25-hydroxyvitamin D3

**Fig. 3:**
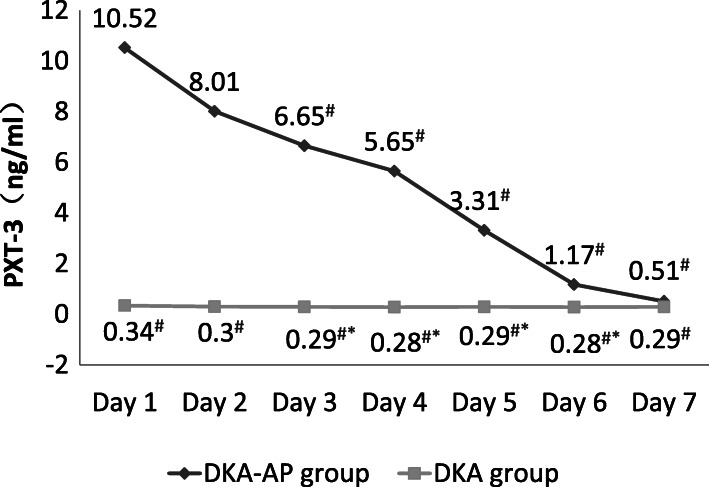
Levels of PTX-3 in DKA-AP and DKA groups. ^#^, compared with the level on day 1 after admission, *P* < 0.05; ^*^, compared with DKA group, *P* < 0.05. PTX-3, PTX-3, pentraxin-3

**Fig. 4:**
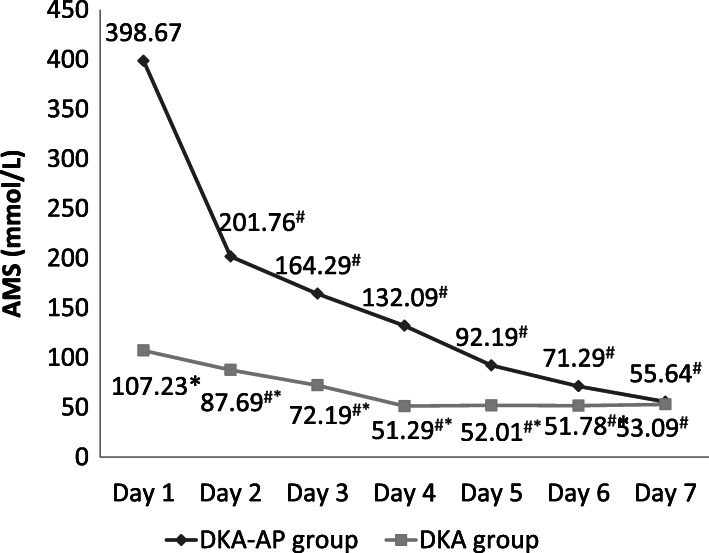
Levels of AMS in DKA-AP and DKA groups. ^#^, compared with the level on day 1 after admission, *P* < 0.05; ^*^, compared with DKA group, *P* < 0.05. AMS, amylase

The Pearson correlation results showed that on the first day after admission, there were positive relativity between the levels of PCT (*r* = 8.321, *P* < 0.05), 25(OH)D (*r* = 9.182, *P* < 0.05), PTX-3 (*r* = 8.132, *P* < 0.05) or AMS (*r* = 7.121, *P* < 0.05) and severity of pancreatitis complicated by diabetic ketoacidosis. In DKA-AP group, the levels of PCT, PTX-3, and AMS were significantly decreased and lower on days 3–7 compared with that on day 1 after admission, and the level of 25(OH)D were significantly increased and higher on days 3–7 compared with that on day 1 after admission (*P* < 0.05) (Figs. 1, 2, 3 and 4). These 4 indexes in the DKA-AP group returned to normal levels on day 7 (Figs. 1, 2, 3 and 4). In DKA group, the levels of PCT, PTX-3, and AMS were significantly decreased on days 2–7 compared with day 1 after admission, and the level of 25(OH)D were significantly increased on days 2–7 compared with day 1 after admission (*P* < 0.05) (Figs. 1, 2, 3 and 4). These 4 indexes in the DKA group returned to normal levels on day 3 (Figs. 1, 2, 3 and 4).

The levels of PCT, PTX-3, and AMS in the DKA-AP group were significantly higher than those in the DKA group on days 1–6, and the level of 25(OH)D were significantly lower than that in DKA group (*P* < 0.05) (Figs. 1, 2, 3 and 4).

### The clinical outcomes between DKA-AP and DKA groups

The duration of hospital stay, patients of ICU care, duration of ICU stay, and cost in the DKA-AP group were all higher compared with the DKA group (*P* < 0.05) (Table 3). There was no significant difference in mortality rate between DKA-AP and DKA groups (*P* < 0.05) (Table 3).

**Table 3:** The clinical outcomes between DKA-AP and DKA groups

Group	Duration of hospital stay, M (P_25_, P_75_), d	Patients of ICU care, n (%)	Duration of ICU stay, M (P_25_, P_75_), d	Mortality, n (%)	Cost, M (P_25_, P_75_), ten thousand yuan
DKA-AP (*n* = 37)	13 (9, 16)	20 (54.05)	4 (3, 5)	2 (5.41)	2.91 (1.98, 3.92)
DKA (*n* = 161)	10 (7, 16)	55 (34.16)	1 (0, 3)	3 (1.86)	1.87 (1.21, 2.92)
χ^2^/Z	24.516	5.063	12.691	1.592	5.762
*P*	< 0.001	0.024	< 0.001	0.229	0.016

*ICU* Intensive care unit

## Discussion

This retrospective study explored the association between potential biomarkers (PCT, 25(OH)D, PTX-3, and AMS) and diabetic ketoacidosis complicated by pancreatitis. The results showed that PCT, 25(OH)D, PTX-3, and AMS were associated with the severity of diabetic ketoacidosis with pancreatitis, which might be potential predictors to guide clinical diagnosis.

Diabetic ketoacidosis is a critical disease, and if complicated by pancreatitis, the disease severity will aggravate leading to poor prognosis. Early diagnosis is very important for these patients. However, effective indexes that could accurately assess the condition of diabetic ketoacidosis complicated by pancreatitis is still lack. The present retrospective study analyzed the general information and some clinical parameters of patients. We found that blood levels of creatine, HbA_lc_, BUN, HDL, AST, ALT, CO_2_CP, glucose, potassium, ketones, hs-TnT, BNP, TG, TC, lactate, lactate and pH, and frequency of diabetic ketoacidosis, type of diabetes, duration of diabetes, vascular complications rates, and abdominal pain rates were significantly different among DKA-AP, DKA and control groups. This result suggested that well-controlled blood glucose levels are of great significance for diabetic patients, which could reduce the incidence of diabetic ketoacidosis with pancreatitis. There was a significant correlation between diabetic ketoacidosis and pancreatitis. In the present study, 18.69% of diabetic ketoacidosis patients were with pancreatitis, which is consistent with a previous report [3]. Abdominal pain is a common symptom in patients with diabetic ketoacidosis, and it also occurs in patients with pancreatitis. Thus, it is difficult to diagnose diabetic ketoacidosis complicated by pancreatitis only rely on a single symptom of abdominal pain.

Diabetic ketoacidosis complicated by pancreatitis is detrimental for patients, which need early diagnosis and treatment. We explored whether PCT, 25(OH)D, PTX-3, and AMS could be potential biomarkers for diabetic ketoacidosis with pancreatitis. PCT is a kind of protein, and occurrences of multiple organ failure, sepsis, serious bacterial, and fungal infections are usually concomitant with elevated levels of PCT [5]. Guidelines reported that PCT levels could be an indicator of infectious: PCT < 0.05 μg/L in healthy people; 0.30 μg/L > PCT > 0.05 μg/L in less than 10% healthy people, chronic disease patients, and old people; PCT > 0.25 μg/L in patients with obvious bacterial infections; > 0.50 μg/L in sepsis patients [5]. Wang et al. [14] suggested that PCT, CRP, and endotoxin were all elevated in patients with acute pancreatitis, and their levels are related to the disease severity and they are independent risk factors for acute pancreatitis. Another study showed that PCT level is significantly increased in patients with diabetic ketoacidosis [6]. Futhermore, Blanchard et al. [15] indicated that PCT levels were different between episodes with and without proven bacterial infection in diabetic ketoacidosis patients. However, there is no conclusive evidence regarding the applicability of PCT in diabetic ketoacidosis or diabetic ketoacidosis with pancreatitis.

Vitamin D regulates calcium and phosphorus metabolism to stabilize blood calcium, as well as pancreatic β-cell function and insulin sensitivity. In addition to these effects, vitamin D also plays an important role in immune response, inflammation, and metabolic syndrome. Gupta et al. [16] reported that the diabetes incidence in people with vitamin D deficiency is 1.41 times higher than those with enough vitamin D, indicating that vitamin D could decrease the incidence of diabetes. The 25(OH)D level is significantly lower in T1D patients, especially those with diabetic ketoacidosis [7]. The incidence of T2D will decrease by 4% if serum 25(OH)D level increases by 10 μg/L [17]. Liu et al. [18] also suggested that diabetic ketoacid patients have lower 25(OH)D levels. Feng et al. [8] confirmed that 25(OH)D levels are lower in patients with moderate-to-severe acute pancreatitis which predict a worse prognosis. There is no study exploring the association of 25(OH)D and diabetic ketoacidosis with pancreatitis.

PTX-3 is a non-specific inflammatory factor and the first human long pentamer, whose genetic localization is in the same superfamily as CRP. PTX-3 can serve as an independent predictor of systemic infections such as sepsis, as well as the disease severity [9]. The study adopted by Merk [10] suggested that in the early stages of pancreatitis PTX-3 concentration is rapidly increased and the severity of pancreatitis could distinguish base on PTX-3 levels. Furthermore, Wang [4] indicated that PTX-3 could be an important indicator for early diagnosis and disease evaluation in pancreatitis. PTX-3 might be a better predictor of prognosis than serum amyloid A and CRP [10].

AMS is composed of isoenzymes, salivary enzyme type (S type), and pancreatic enzyme type (P-type), which are the most important hydrolytic amylases. The P-type amylase is secreted by the pancreas and the S-type amylase is secreted by the salivary glands. If the pancreas is affected by inflammation or other diseases, pancreatic cells will increase cell permeability and necrosis, triggering an increased blood level of AMS. Most patients with pancreatitis have elevated blood amylase for 2–12 h after symptom onset, which returned to normal levels over 3–5 days. Xu suggested that the AMS level does not linearly correlate with the severity of pancreatitis [19]. Zhong [11] found that the AMS level was associated with diabetic ketoacid.

However, there is a lack of clinical studies on investigating the association between PCT, 25(OH)D, PTX-3, and AMS and diabetic ketoacidosis complicated by pancreatitis. Our results showed that on the first day after admission, the levels of PCT, PTX-3, and AMS in the DKA-AP group were significantly higher compared with DKA and control group, while 25(OH)D levels were lower. Meanwhile, the blood levels of PCT, 25(OH)D, PTX-3, and AMS were correlated with the severity of diabetic ketoacidosis with pancreatitis. This evidence indicated that PCT, 25(OH)D, PTX-3, and AMS could be indicators for diabetic ketoacidosis with pancreatitis.

We also found that PCT, PTX-3 and AMS levels in the DKA-AP group showed a downward trend and 25(OH)D levels showed an upward trend after 3 days after admission. PCT, PTX-3, and AMS levels in most patients returned to normal levels within 7 days. In the DKA group, the changes of PCT, 25(OH)D, PTX-3, and AMS levels was transient, and the levels returned to normal values in most patients within 3 days. Moreover, the levels of PCT, 25(OH)D, PTX-3, and AMS levels were significantly different between DKA-AP and DKA groups on days 1–6 after admission. These results suggested that to a certain extent, levels of PCT, 25(OH)D, PTX-3, and AMS can reflect the disease severity of diabetic ketoacidosis with pancreatitis. According to our clinical experience, diabetic ketoacidosis with pancreatitis should be diagnosed by symptoms, predisposing factors, and imaging results. If the first symptom of patients with diabetic ketoacidosis is abdominal pain, nausea and vomiting, and the PCT, 25(OH)D, PTX-3, and AMS levels are significantly changed, clinicians need to distinguish carefully from pancreatitis to avoid missing best chance to treat.

This study compared clinical outcomes between patients in DKA-AP and DKA groups and showed that there was no significant difference in mortality rate. However, patients with diabetic ketoacidosis complicated by pancreatitis had a higher length of hospital stay and ICU stay, elevated rate of ICU care, and increased cost patients compared with patients without pancreatitis diabetic ketoacidosis. It might because that the disease condition of diabetic ketoacidosis with pancreatitis is more complicated and severe, and the altered levels of PCT, 25(OH)D, PTX-3, and AMS might also contribute to it.

Due to the limited time and small sample size of this study, we suggest that higher-quality prospective studies are needed to confirm our conclusions. In the future, establishing integral discriminatory and mathematical models based on the conclusions might facilitate the diagnosis of diabetic ketoacidosis with pancreatitis.

## Conclusions

Enough attention should be paid to diabetic ketoacidosis complicated by pancreatitis to avoid serious consequences. The levels of PCT, 25(OH)D, PTX-3, and AMS are correlated with diabetic ketoacidosis with pancreatitis, which are closely related to the disease severity.

## Data Availability

The datasets generated and analyzed during the current study are available from the corresponding author on reasonable request.

## Abbreviations

ALT: Alanine aminotransferase
AMS: Amylase
APACHE II: Acute physiology and chronic health evaluation II
AST: Aspartate aminotransferase
BMI: Body mass index
BNP: Brain natriuretic peptide
BUN: Blood urea nitrogen
CRP: C-reactive protein
CO_2_CP: Carbondioxide combining power
HbA_lC_: Hemoglobin A_1C_
HDL: High-density lipoprotein
hs-cTnT: High sensitivity cardiac troponin testing
ICU: Intensive care unit
IL: Interleukin
LDL: Low-density lipoprotein
PCT: Procalcitonin
PTX-3: Pentraxin-3
P type: Pancreatic enzyme type
SD: Standard deviation
S type: Salivary enzyme type
TC: Total cholesterol
TG: Triacylglyceride
25(OH)D: 25-hydroxyvitamin D3.
